# Integrating single-cell and spatial transcriptomics reveals the cellular heterogeneity of vestibular schwannoma

**DOI:** 10.1038/s41698-025-01028-y

**Published:** 2025-07-08

**Authors:** Wenqi Dong, Yuchen Jin, Lingkang Dong, Yumeng Jiang, Zhuangzhuang Li, Maoxiang Xu, Jingjing Wang, Feng Liu, Dongzhen Yu

**Affiliations:** 1 Shanghai Key Laboratory of Sleep Disordered Breathing, Department of Otolaryngology-Head and Neck Surgery, Otolaryngology Institute of Shanghai Jiao Tong University, Shanghai Sixth People’s Hospital Affiliated to Shanghai Jiao Tong University School of Medicine, Shanghai, China; 2 Department of Otolaryngology, Sun Yat-sen Memorial Hospital, Sun Yat-sen University, Guangzhou, China; 3 Department of Otolaryngology-Head and Neck Surgery, Shanghai Fourth People’s Hospital, and School of Medicine, Tongji University, Shanghai, China

**Keywords:** Cancer microenvironment, Head and neck cancer

## Abstract

Vestibular schwannoma (VS) is a benign tumor that can result in significant neurological and otological complications. The mechanisms underlying its development and spatial heterogeneity remain poorly understood. In this study, we analyzed single-cell RNA sequencing (scRNA-seq) data from three previously published vestibular schwannomas (VS1-3), along with spatial transcriptomics data from two additional specimens (VS_S1-2). Our results identified a VEGFA-enriched Schwann cell (SC) subtype in scRNA-seq data, which was validated by spatial transcriptomics. This subtype also exhibited a significant positive correlation with *NOV*^hi^ SCs expression. These cells were centrally localized within tumor tissue. Furthermore, spatial analysis provided new insights into SC-stromal cell interactions, and we constructed a preliminary cellular atlas of VS tissues, enhancing our understanding of tumor growth dynamics.

## Introduction

Vestibular schwannoma (VS) is a common intracranial space-occupying lesion, accounting for 7%–12% of all intracranial tumors and 80%–95% of cerebellopontine angle tumors^1,2^. Accumulating evidence indicates that VS is a slow-growing benign tumor originating from Schwann cells (SCs) of the vestibulocochlear nerve sheath^2,3^. Common presenting symptoms include hearing loss with tinnitus (24.8%), isolated hearing loss (20.2%), and hearing loss accompanied by tinnitus and balance disorders (15.6%)^4^. Current treatment strategies for VS encompass surveillance (wait-and-scan), radiotherapy, microsurgery, or a combination of these approaches^2^. However, each strategy has its own limitations^5^. For tumors larger than 2.5 cm, fewer than 5% of patients retain functional hearing, whereas 50% of those undergoing gross total resection develop permanent partial or complete facial nerve paralysis^6,7^. To improve clinical outcomes, further investigation into the molecular drivers underlying VS pathogenesis is imperative.

The application of single-cell RNA sequencing (scRNA-seq) to patient tumors has provided opportunities to uncover multiple cell populations and investigate dynamic cell-state fluctuations within tissues^8–10^. In our previous study^11^, we identified five Schwann cell (SC) subtypes—*GFRA3*^+^ SCs, *VEGFA*^+^ SCs, *FOSB*^+^ SCs, *PMP2*^hi^_SCs and *PRX*^+^ SCs—each characterized by distinct gene expression profiles. *PRX*^+^ SCs exhibited fewer tumorigenic properties due to their expression of *NCMAP* and *MAG*—markers highly expressed in mature myelinating Schwann cells. Other clusters expressing elevated levels of tumor-related markers, for example, *SAMHD1*, *GFRA3* and *VGLL3*, were classified as SCs with neoplastic potential. Furthermore, our study demonstrated that tumor-associated fibroblasts promote tumorigenesis through the integrin–*IGF*/*MDK* signaling axis^11^. Chu et al. applied scRNA-seq to sporadic VS tissues and revealed functional heterogeneity among SC clusters, including roles in neuronal survival and axon regeneration^12^. Their work further highlighted the critical contribution of stromal cells to tumor proliferation and growth^12^. Additionally, injury-like tumor phenotypes were associated with larger tumor size and *CSF1*-mediated myeloid cell recruitment by VS-SCs, as demonstrated through deconvolution analysis and scATAC-seq^13^. Moreover, gene expression profiles of tumor-driving cell clusters were leveraged to refine growth classification in bulk RNA sequencing data, with normal great auricular nerves serving as a control^14^.

The lack of spatial mapping obscures the hierarchical mechanisms governing Schwann cell positioning and identity acquisition. Spatial transcriptomics, as an emerging technology, provides high-resolution mapping of gene expression across tissue sections, circumventing limitations inherent in tissue dissociation^15–17^. In this study, we integrated scRNA-seq with spatial transcriptomics in adult vestibular schwannoma tissues to investigate tumor cell phenotypic heterogeneity and progression. We analyzed cellular diversity in five VS patients, including three with scRNA-seq profiles (VS1–VS3, from our prior cohort^11^) and two with spatial transcriptomics data. Within this integrated dataset, we focused on SC subsets exhibiting tumor-associated molecular signatures. Our study delineates VS spatial heterogeneity, revealing intercellular signals that regulate SC positioning and functional states. This multimodal dataset serves as a foundation for probing tumor-immune dynamics and therapeutic targets.

## Results

### Single-cell landscapes reveal major cell types from human in the vestibular schwannoma group

**Fig. 1:**
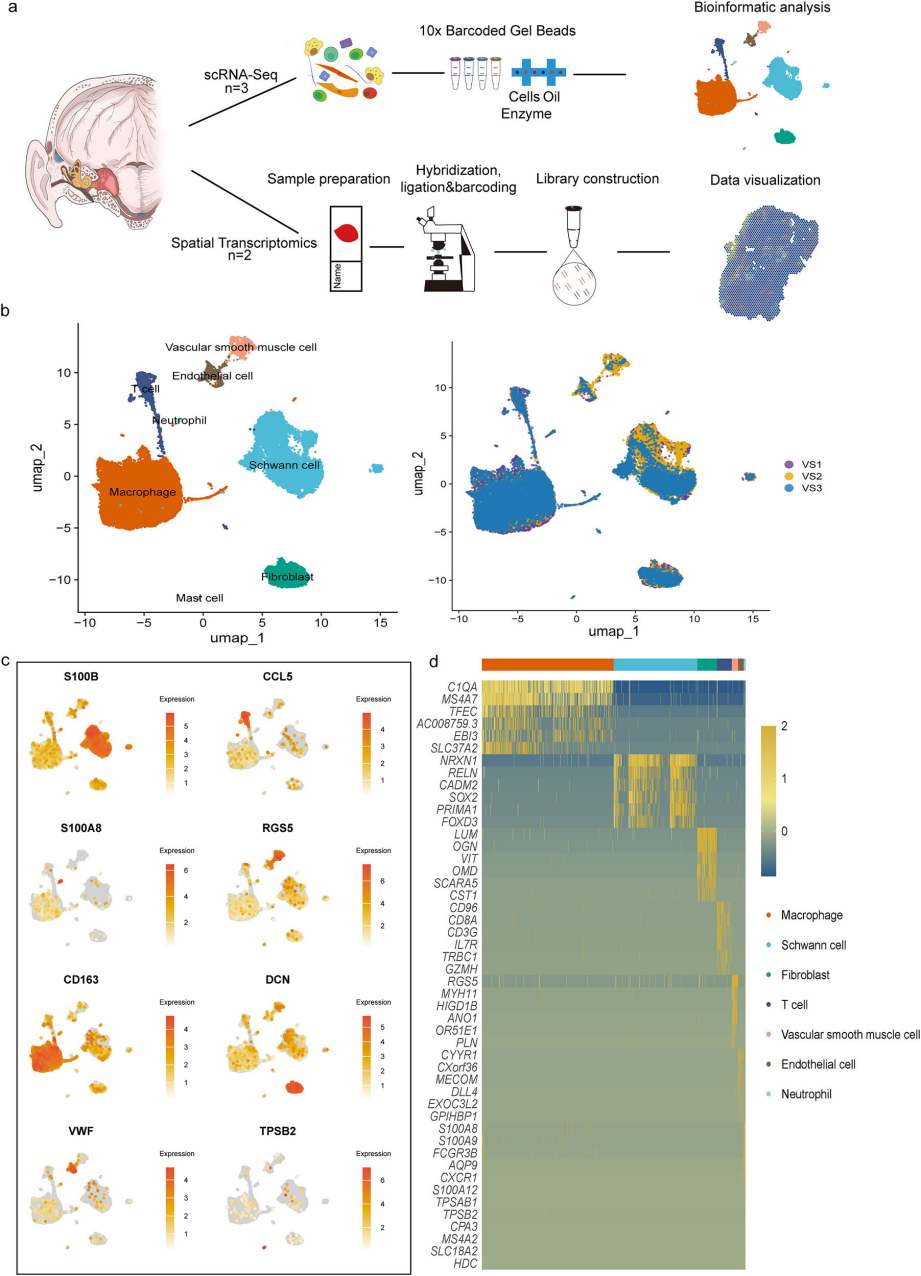
Single-cell transcriptomic profiling of vestibular schwannoma. **a** Schematic overview of the study design. Surgical specimens from five VS patients were subjected to single-cell RNA sequencing (scRNA-seq) or spatial transcriptomics to characterize transcriptional and spatial heterogeneity. **b** Uniform Manifold Approximation and Projection (UMAP) plot of integrated scRNA-seq data, color-coded by annotated cell types across VS tumor tissues (*n* = 3) (left) and patient origin (right). **c** Feature plots visualizing lineage-defining gene expression gradients (scaled mean expression), including Schwann cell markers (*S100B*), immune cell signatures (*CCL5, S100A8, CD163, TPSB2*), and stromal markers (*RGS5*, *DCN*, *VWF*). **d** Heatmap of differentially expressed genes (DEGs) across cell clusters (row-scaled z-scores). Lineage-specific gene modules are highlighted on the left.

To elucidate the cellular architecture of vestibular schwannoma, we integrated the scRNA-seq data from three VS tumors tissues (Fig. 1a). Unsupervised clustering identified eight major cell populations, annotated using canonical lineage-specific markers from published studies: Schwann cell (*SOX2*, *PMP2*, *S100B*)^18,19^; immune lineages including macrophage (*PTPRC*, *CD68*, *CD74*)^20,21^, T cell (*CCL5*, *TRBC2*, *CD3E*)^22^, neutrophil (*S100A9*, *S100A8*)^23^, mast cell (*TPSB2*, *CPA3*, *MS4A2*); as well as stromal components comprising fibroblast (*DCN*, *LUM*, *CYP1B1*), vascular smooth muscle cell (*RGS5*, *ACTA2*, *TAGLN*), and endothelial cell (*PLVAP*, *VWF*, *FLT1*)^24^(Fig. 1b, c). To facilitate future investigations, we systematically compiled a reference panel of subpopulation-specific markers across cellular lineages, providing a foundation for probing VS heterogeneity (Fig. 1d).

### Differential gene expression and Schwann cell subcluster dynamics in vestibular schwannoma

As Schwann cells constitute the predominant neoplastic population in vestibular schwannoma (VS) and undergo dynamic transcriptional remodeling during tumorigenesis, we performed an analysis of SC subpopulations in VS tumors. Through clustering, we identified five subclusters within the scRNA-seq data: *NOV*^hi^ SC, *JUNB*^*+*^ SC, *PMP2*^*+*^ SC, *VEGFA*^*+*^ SC, and *PRX*^*+*^ SC (Supplementary Fig. 2a). Differential expression analysis across subclusters identified lineage-defining markers, with the top four discriminative genes per subtype visualized via dot plot (Supplementary Fig. 2b).

**Fig. 2:**
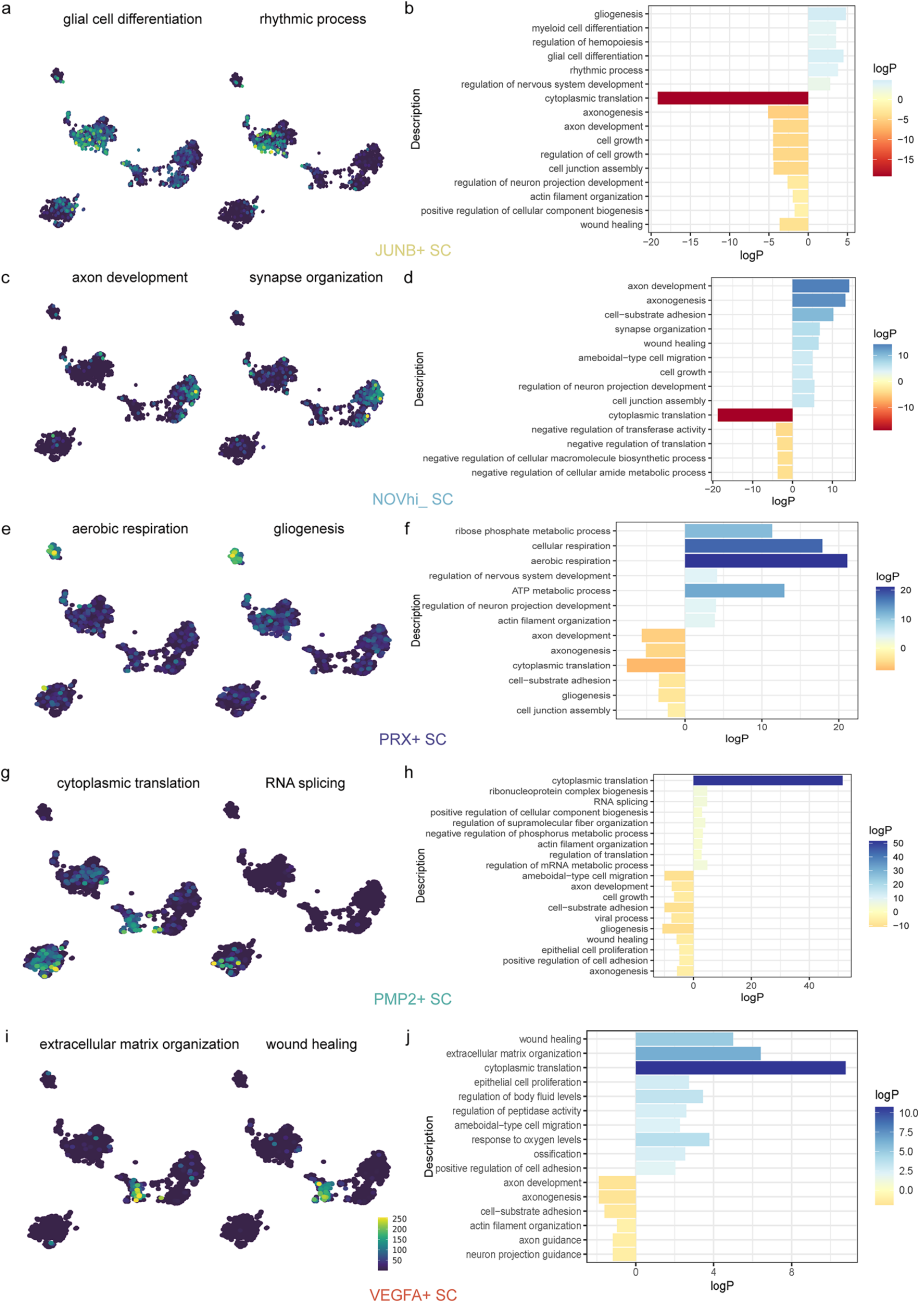
Gene ontology (GO) analysis of five subclusters of Schwann cells in vestibular schwannoma from scRNA-seq. **a**, **b** Analysis of biological process (BP) based on DEGs of *JUNB*^+^ SC from scRNA-seq data. **c**, **d** Analysis of BP based on DEGs of *NOV*^hi^ SC from scRNA-seq data. **e**, **f** Analysis of BP based on DEGs of *PRX*^+^ SC in VS. **g**, **h** Analysis of BP based on DEGs of *PMP2*^+^ SC. **i**, **j** Analysis of BP based on DEGs of *VEGFA*^+^ SC in VS.

Gene Ontology (GO) enrichment analysis delineated distinct functional specializations across Schwann cell subclusters (Fig. 2). The *JUNB*^+^ SC subtype exhibited pronounced enrichment in developmental regulation pathways, including gliogenesis, myeloid differentiation, and nervous system development (Fig. 2a, b), suggesting a role in microenvironmental modulation. *NOV*^hi^ SCs were associated with axonal morphogenesis and synaptic organization (axon development, synapse organization; Fig. 2c, d). *PRX*^+^ SCs demonstrated dual metabolic and structural roles, with predominant enrichment in energy metabolism (ribose phosphate metabolic process, aerobic respiration) and cytoskeletal organization (actin filament organization; Fig. 2e, f). *PMP2*^+^ SCs showed robust engagement in translational machinery (cytoplasmic translation, ribonucleoprotein complex biogenesis; Fig. 2g, h), indicative of heightened biosynthetic activity. Strikingly, *VEGFA*^+^ SCs emerged as key stromal interactors, displaying angiogenic (extracellular matrix organization, wound healing) and proliferative (epithelial cell proliferation) signatures alongside protease regulation (Fig. 2i, j).

### Spatial transcriptome combined with scRNA-seq revealed regionalisation of vestibular schwannoma samples

**Fig. 3:**
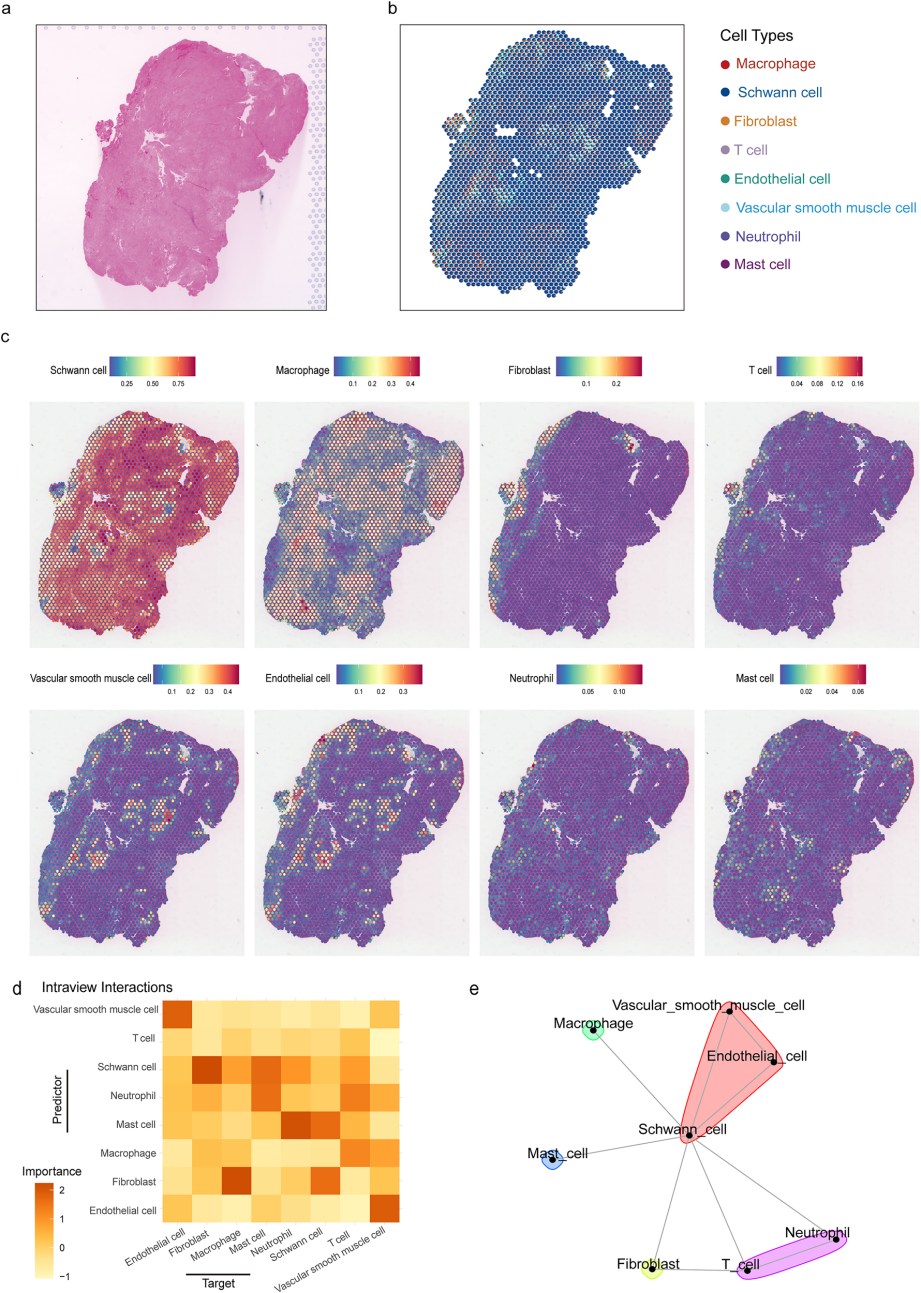
Spatial transcriptomics revealed the transcriptional architecture of VS_S1. **a** H&E staining of VS_S1 sample. **b** Description of ST data using cell-type deconvolution in VS_S1. **c** Spatial expression of different cell types in the VS_S1 sample. The color indicates the expression levels of the subgroup genes. **d** Median importance of cell-type abundance in the prediction of abundances of major cell types within a spot in VS_S1. **e** Schematic representation of intraview cell‒cell interaction networks using MISTy within Visium spots in VS_S1.

To investigate the spatial distribution patterns of vestibular schwannoma, we conducted spatial transcriptomics analysis on tumor specimens from two VS patients (Fig. 1a). For the VS_S1 sample, we identified 2294 high-quality spatial spots with a median sequencing depth of 224,536 reads per spot. Histopathological evaluation of hematoxylin-eosin (HE)-stained sections revealed that the VS_S1 specimen predominantly exhibited the Antoni-A histopathological pattern (Fig. 3a). To delineate cellular heterogeneity, we employed RCTD^25^ to deconvolve cell-type abundance patterns across VS tissue sections. This spatial deconvolution approach integrated single-cell RNA sequencing reference profiles with spatial transcriptomics data, enabling systematic identification of major cell populations within spatially resolved domains. In the VS_S1 spatial transcriptomics dataset, we consistently identified major cell types (Fig. 3b, c). Notably, the abundance scores of macrophages, fibroblasts, vascular smooth muscle cells (VSMCs), endothelial cells, T cells, neutrophils and mast cells were comparatively lower – an expected observation given that the VS_S1 sample represents whole tumor tissue and its Antoni-A histopathological pattern.

To further validate spatial dependencies, we assessed the predictive capacity of cellular neighborhood contexts—characterized by adjacent regional cell type composition—on spot-level major cell type abundance across three spatial scales: intra-spot (~ 55 μm), proximal view (~ 200 μm; 5-spot radius), and distant view (~ 3000 μm; 15-spot radius). The analysis revealed that endothelial cells exhibited strong predictive power for VSMCs abundance across all examined spots, reflecting functional interdependencies among vasculature-associated cell types (Fig. 3d and Supplementary Fig. 3a). Schwann cells demonstrated the highest predictive capacity for fibroblast, macrophage, mast cell, neutrophil, and T cell abundance throughout all detection spots. Notably, strong co-localization relationships were observed between fibroblasts and Schwann cells, indicating marked cellular dependency existed between the two cell types. A significant macrophage-fibroblast correlation was identified, consistent with fibroblast-mediated macrophage chemotaxis mechanisms (Fig. 3d). The co-occurrence of VSMCs, endothelial cells, and Schwann cell highlighted their spatial interdependencies, emphasizing the biological significance of vascular-associated cell types with Schwann cell in vestibular schwannoma pathogenesis (Fig. 3e).

**Fig. 4:**
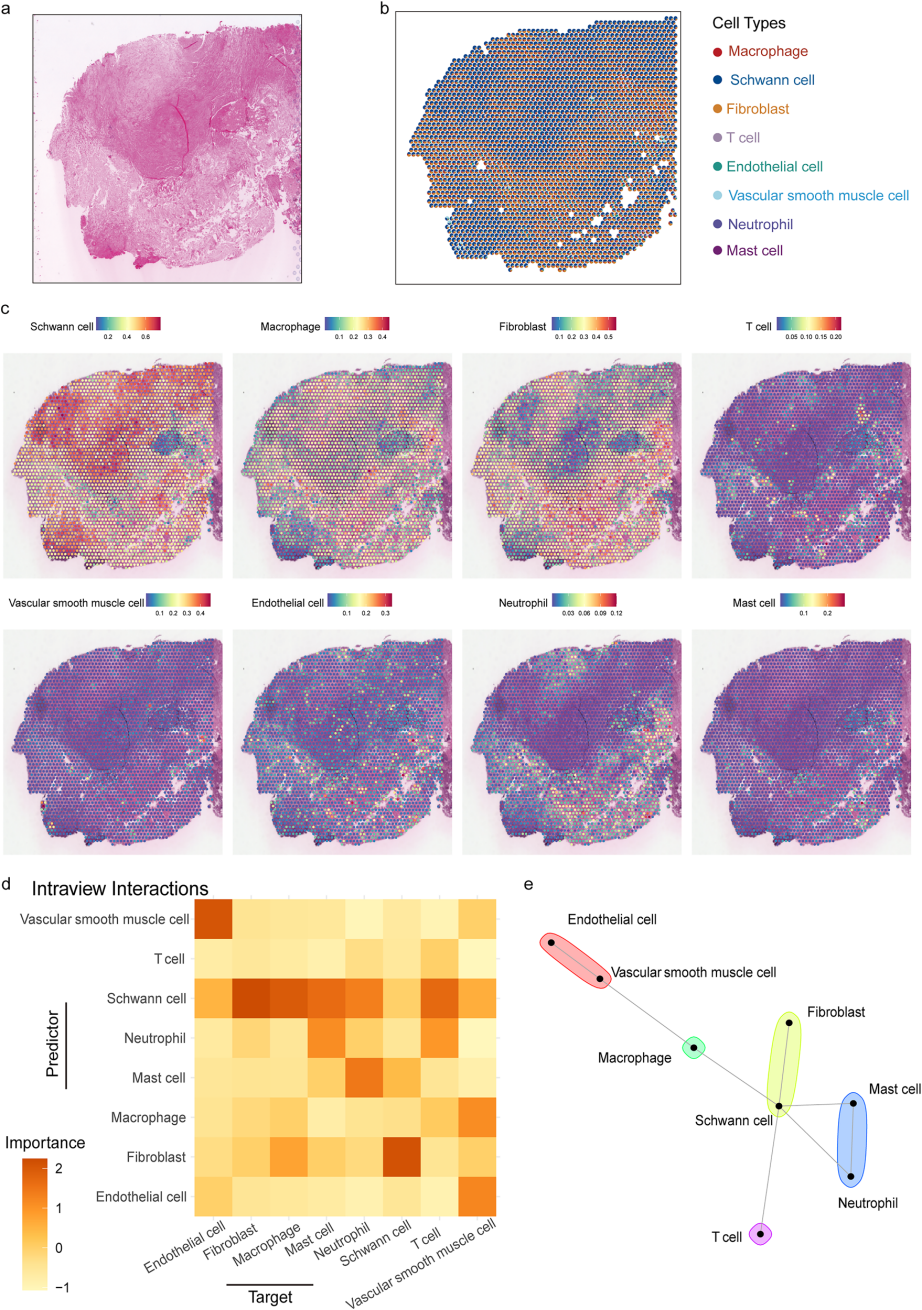
Spatial transcriptomics revealed the transcriptional architecture of VS_S2. **a** H&E staining of VS_S2 sample. **b** Description of ST data using cell-type deconvolution in VS_S2. **c** Spatial expression of different cell types in the VS_S2 sample. The color indicates the expression levels of the subgroup genes. **d** Median importance of cell-type abundance in the prediction of abundances of major cell types within a spot in VS_S2. **e** Schematic representation of intraview cell‒cell interaction networks using MISTy within Visium spots in VS_S2.

In the VS_S2 specimen, spatial transcriptomic profiling identified 2,799 high-quality spots with a median sequencing depth of 179,011 reads per spot. Comparative histopathological evaluation of HE-stained sections revealed co-existing Antoni-A and Antoni-B architectural patterns in the VS_S2 sample (Fig. 4a). Spatial deconvolution analysis of the VS_S2 transcriptomic landscape successfully mapped the distribution patterns of major cellular constituents (Fig. 4b, c). Comparative analysis revealed distinct patterns in VS_S2 versus VS_S1: fibroblasts showed enhanced predictive power for Schwann cell abundance with attenuated macrophage co-localization, potentially attributable to sample-specific pathological architectures (Fig. 4d). Consistent with VS_S1, VS_S2 maintained significant lymphoid-myeloid interaction networks, preserving conserved immune cell interplay and inflammatory niche characteristics (Fig. 4d, e).

### Spatial ecotype characterization revealed multicellular functional niches in Vestibular Schwannoma

The co-localization of Schwann cells with immune and stromal cells underscores the potential of investigating tumor architecture to deepen our understanding of functional roles in tumor cell states. We therefore sought to determine whether vestibular schwannoma harbors spatially organized multicellular communities that extend beyond mere co-occurrence and abundance, which may provide critical insights into the region-specific biological functions within VS. To identify these multicellular communities and their spatial distribution patterns, we employed ISCHIA for regional clustering based on cell type composition across all samples^26^. Following the nomenclature proposed by Khaliq et al., we define these cell-type-specific microenvironments or regionally distinct cellular compositions - collectively referred to as regional cellular architecture - as “spatial ecotypes”^27^.

**Fig. 5:**
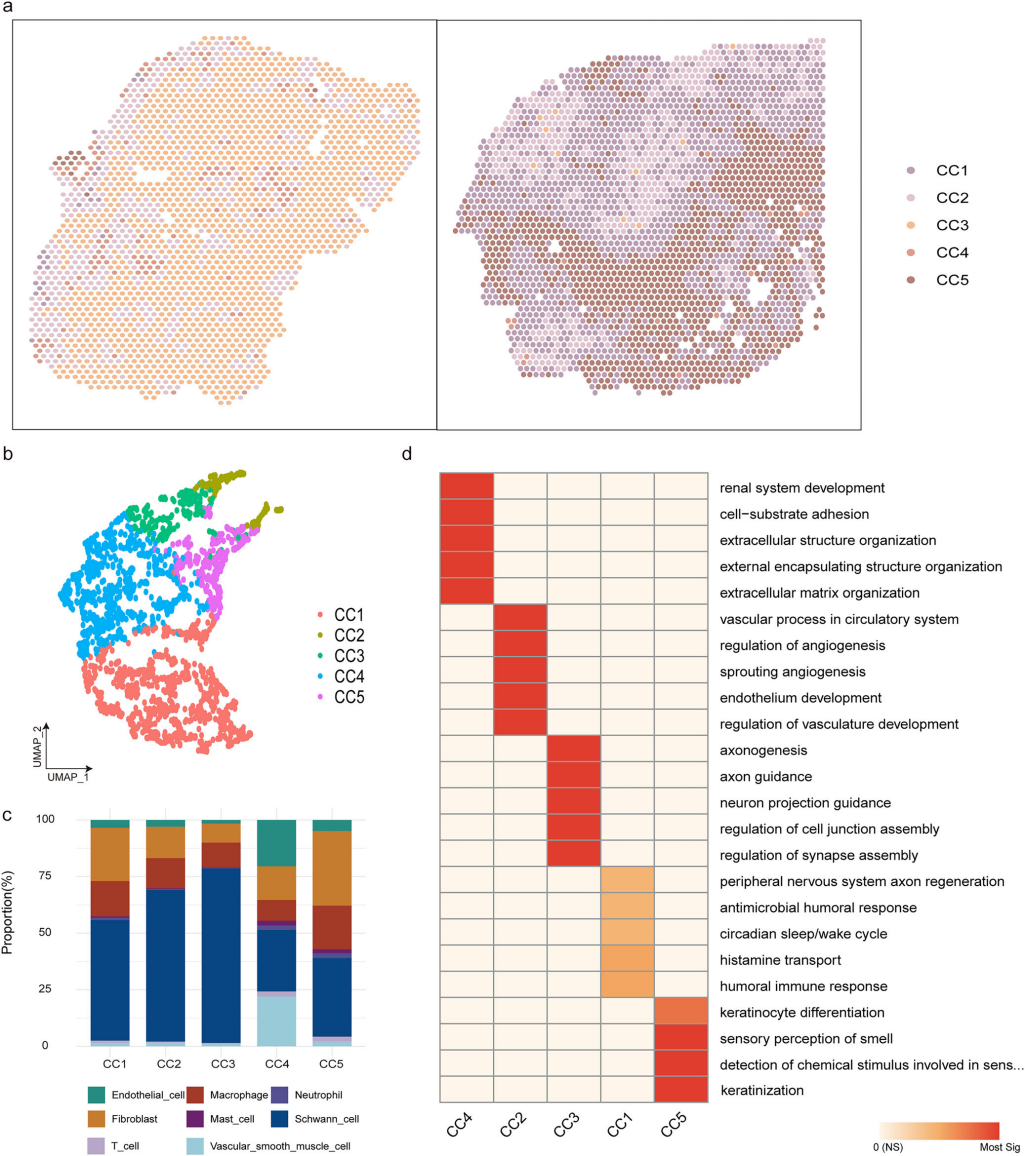
Spatial ecotype characterization revealed multicellular functional niches in Vestibular Schwannoma. **a** Visualization of spatial ecotypes in VS_S1 and VS_S2 samples. **b** UMAP plot of ST spots clustered by cell-type composition-based *k*-means clustering colored by five spatial ecotypes. **c** Bar plots showing cell-type composition of each spatial ecotype. **d** Heatmap of enrichment patterns of five ecotypes.

Through spatial transcriptomic analysis, we identified five transcriptionally defined ecotypes with distinct cellular compositions and functional characteristics (Fig. 5a, b). The CC2 and CC3 ecotypes, predominantly composed of Schwann cells, spatially aligned with Antoni-A regions. In contrast, CC4 and CC5 exhibited composite structures of Schwann cells, fibroblasts, and diverse immune subpopulations, corresponding to the pathological features of Antoni-B regions, while CC1 represented a transition zone with intermediate phenotypic traits (Fig. 5c). Functional enrichment analysis revealed that CC1 was enriched for genes associated with axonal regeneration and neuroprotection; CC2 with vascular system development; CC3 was enriched for genes associated with regulated nervous system development and functional connectivity; CC4 was associated with modulation of the cellular microenvironment and played a critical role in tissue development/repair and CC5 with barrier formation and inflammatory regulation (Fig. 5d). These ecotype-function correlations were further validated by spatial distribution patterns, where CC2 and CC3 predominated in VS_S1 samples with Antoni-A architecture, whereas CC1, CC2 and CC5 were uniformly distributed in VS_S2 samples exhibiting mixed Antoni A and B features (Fig. 5a).

### Architecture of Schwann cells in the vestibular schwannoma with spatial transcriptomics

**Fig. 6:**
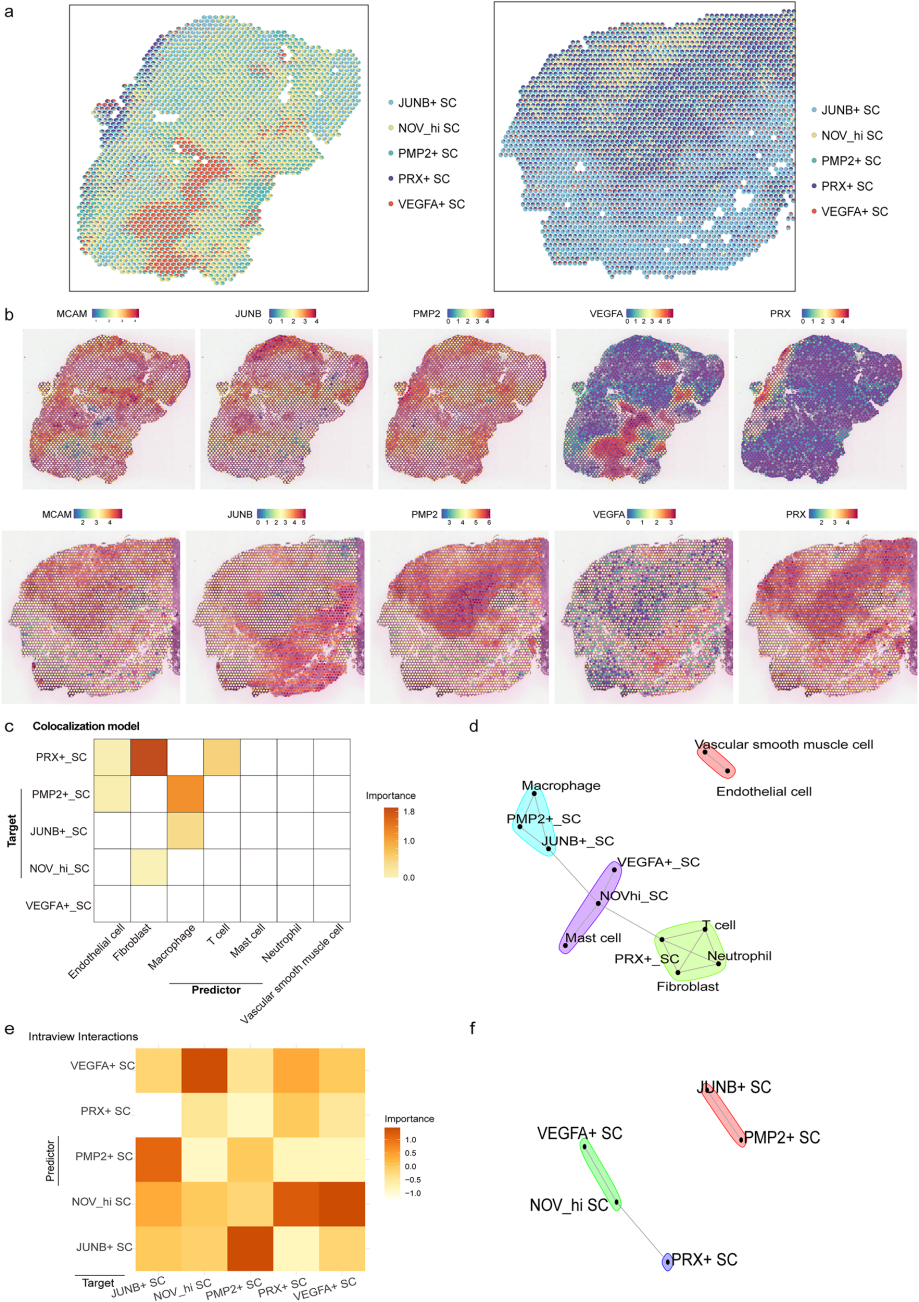
Spatial identification of regional Schwann cell subtypes. **a** Description of SCs ST data using cell-type deconvolution in VS_S1 (left) and VS_S2 (right). **b** Spatial expression of *MCAM*, *JUNB*, *PMP2*, *VEGFA*, *PRX* in VS_S1 and VS_S2 samples. **c** Median importance of the abundance of the other cell types within a spot in the prediction of SCs-state scores in spatial transcriptomics. **d** Schematic representation of intraview cell‒cell interaction networks using MISTy within Visium spots of SCs. **e** Median importance of the abundance of SC subtypes within a spot in the prediction of SCs-state scores in spatial transcriptomics. **f** Schematic representation of intraview cell‒cell interaction networks within SC subtypes.

To identify regionally enriched Schwann cells, we first annotated and spatially mapped molecularly distinct Schwann cell subtypes that were identified based on our scRNA-seq reference. In the VS_S1 sample, *JUNB*^*+*^ SCs exhibited higher expression in certain regions but relatively lower expression across most of the sample. *PMP2*^+^ SCs and *NOV*^hi^ SCs displayed more widespread expression. *PRX*^+^ SCs had high expression in special, localized regions, surrounding the expression region of fibroblasts. *VEGFA*^+^ SCs were predominantly located in the core region of the tissue, suggesting focal gene activity that may be biologically significant in these regions (Fig. 6a, b). In the VS_S2 sample, the areas of high expression of *NOV*^hi^ SCs were concentrated farther away from the areas of predominant expression with fibroblasts. *PMP2*^+^ SCs and *JUNB*^+^ SCs revealed distinct expression regions that coincided with structural features in the stained tissue, suggesting that their activity may be related to particular tissue morphology. *PRX*^+^ SCs and *VEGFA*^+^ SCs also displayed localized expression, and with the tissue background, it became more apparent that *VEGFA* was concentrated in discrete regions, possibly indicating areas of angiogenesis or vascular involvement (Fig. 6a, b).

Through spatial transcriptomic modeling, we investigated the spatial associations between Schwann cell subtypes and the abundance of other major cell populations. Our analysis revealed that *PRX*^+^ SCs distribution was optimally predicted by fibroblast density within the tissue spot. The expression levels of *PMP2*^+^ SC and *JUNB*^+^ SC markers showed the strongest correlations with intra-tissue macrophage presence. *NOV*^hi^ SCs subtype markers were primarily influenced by fibroblast distribution in local tissue regions (Fig. 6c). Notably, *VEGFA*^+^ SCs abundance exhibited no significant correlation with non-Schwann cell populations (Fig. 6c, d). Further analysis of intra-subtype and its five-spot radius neighborhood co-localization patterns demonstrated a significant positive correlation between *VEGFA*^+^ SCs abundance and *NOV*^hi^ SCs expression (Fig. 6e, f and Supplementary Fig. 3b).

### Communication between fibroblasts and SCs might facilitate vestibular schwannoma progression from diverse aspects

**Fig. 7:**
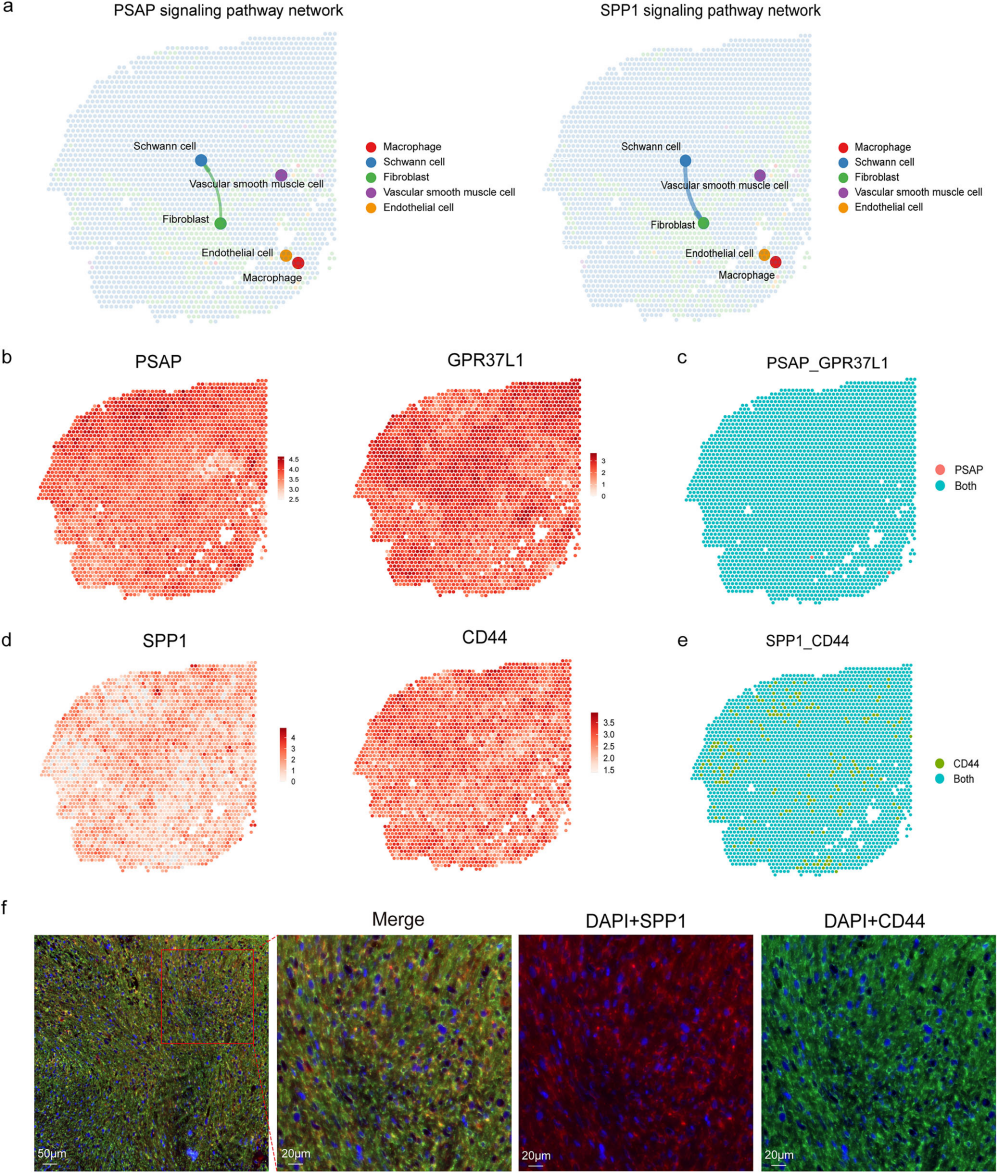
Cellular ligand and receptor interactions in vestibular schwannoma. **a** Spatial images of *PSAP* and *SPP1* signaling pathway network, the thickness of lines represents the strength of signals. **b**, **c** Spatial plots showing the expression of *PSAP*, *GPR37L1*(**b**) and *PSAP*/*GPR37L1*(**c**). **d**, **e** Spatial plots showing the expression of *SPP1*, *CD44* (**d**) and *SPP1*/*CD44* (**e**). **f** Representative images of multiplex immunofluorescence staining indicating *SPP1*^+^ and *CD44*^+^ cells in VS. Scale bar, 20 and 50 μm.

Cell-cell interactions provide deeper insights into organ development, homeostasis, and disease mechanisms^28–30^. We investigated the cellular interactions among different cell types in the VS_S2 sample using CellChat V2. We observed *PSAP*/*GPR37L1* and *SPP1*/*CD44* ligand-receptor pairs are significantly enriched between Schwann cells and fibroblasts and characterized their spatial expression patterns (Fig. 7a–e). *PSAP* is a highly conserved glycoprotein considered a neurotrophic factor, known to protect neural cells from damage^31,32^. As a receptor for *PSAP*, *GPR37L1* plays a role in the activation of satellite cells and neurons. *SPP1*, also known as osteopontin, is an O-glycosylated phosphoprotein produced by various tissues and cells and secreted into body fluids^33^. Its ability to interact with multiple, widely expressed cell surface receptors allows it to play an active role in a range of physiological and pathological processes, including inflammatory, degenerative, autoimmune, and oncologic diseases^34^. Previous studies have shown that *SPP1* expression level significantly increases at injury sites and that it specifically promotes the outgrowth of injured motor axons, but not sensory neurons^35^. Our research demonstrated that the *SPP1*/*CD44* pathway was activated in vestibular schwannoma tissues, aligning with findings from earlier studies^34,35^. Spatial transcriptomics mapping and co-localization analysis revealed the expression and co-localization of the *PSAP*/*GPR37L1* and *SPP1*/*CD44* ligand-receptor pairs within the VS_S2 sample (Fig. 7b–e). We then performed multiplex immunofluorescence staining using specific antibodies against *SPP1* and *CD44*. We confirmed the co-localization of the ligand-receptor pair (Fig. 7f). These findings suggested that the interactions of these ligand-receptor pairs might play crucial roles in the occurrence and development of vestibular schwannoma.

**Fig. 8:**
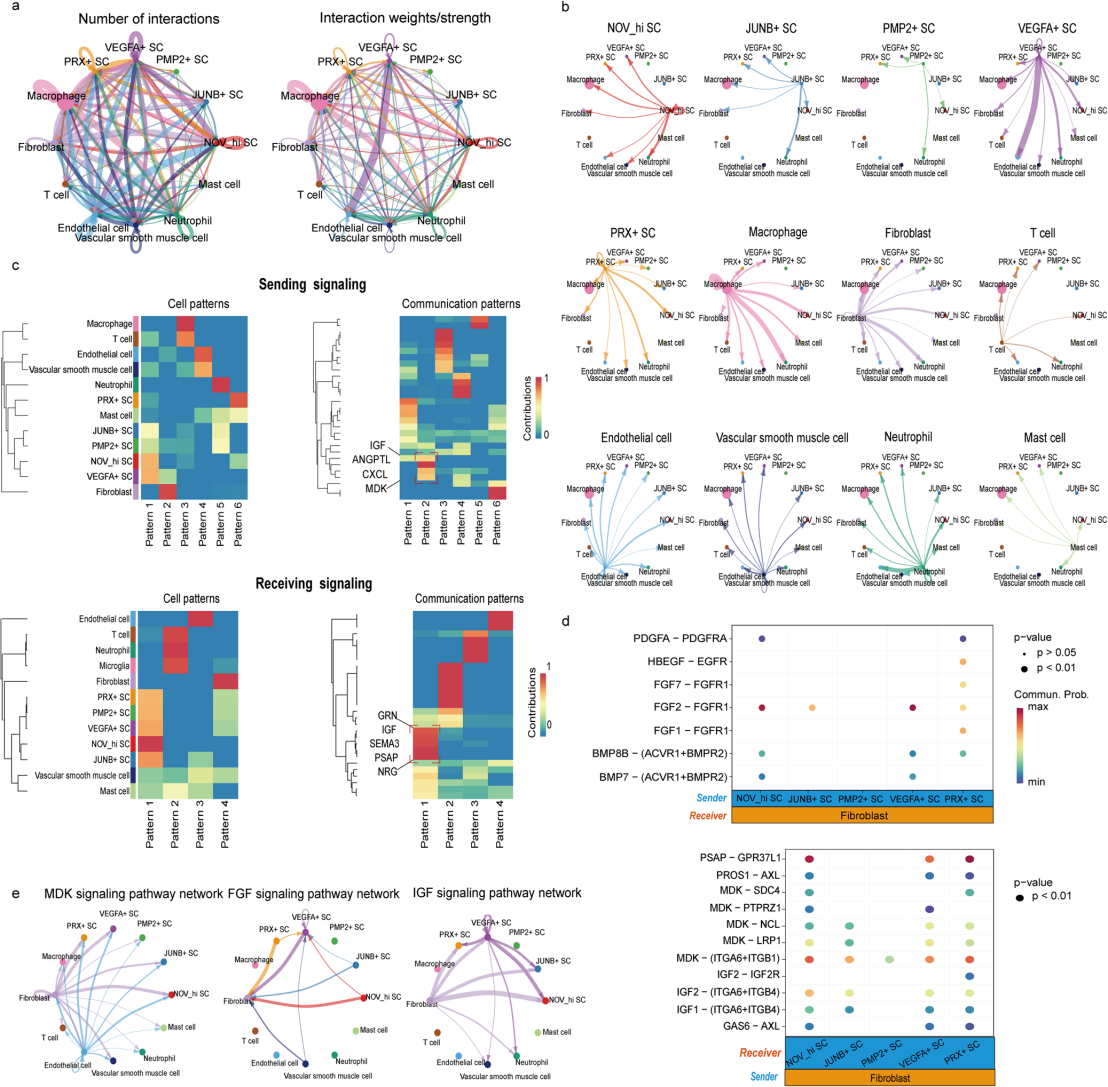
Cell communication prediction. **a** Circle plot depicting the number of interactions and their weight/strength among various cell types. **b** Detailed visualization of ligands broadcasted by each major cell population. **c** Heatmap illustrating the receiving and sending signal patterns of all cell types as identified by CellChat analysis. **d** Ligand-receptor communication network between fibroblasts and the five Schwann cell subtypes predicted using scRNA-seq data. **e** Circle plot demonstrating the inferred *MK*, *FGF* and *IGF* signaling networks among different cell types.

The development of tumors heavily relies on their close interactions with the surrounding cellular environment^36–38^. Interestingly, significant differences were observed between Schwann cells in terms of their interaction intensity with fibroblasts. As a result, we performed an analysis of cell-cell communication to explore the interactions between five Schwann cell subtypes and fibroblasts. We identified many ligand-receptor pairs, demonstrating extensive molecular interactions between five different Schwann cell subtypes and fibroblasts (Fig. 8a, b). As Fig. 8c shows, *NOV*^*hi*^ SCs, *JUNB*^*+*^ SCs, *PMP2*^*+*^ SCs, *VEGFA*^*+*^ SCs, and *PRX*^*+*^ SCs primarily accepted signals from *GRN*, *IGF*, *SEMA3*, *PSAP* and *NRG* molecules for receiving signaling. Regarding sending signals, fibroblasts mainly produced signals involving *IGF*, *ANGPTL*, *CXCL*, and *MDK* (Fig. 8c). Additionally, fibroblasts were identified as central cells in several cell communication networks, including the *FGF*, *PSAP*, *MDK*, and *IGF* signaling pathways (Fig. 8d–e). The *FGF*/*FGFR* signaling network impacts tumorigenesis through a variety of mechanisms, such as the dysregulation of cell signaling, promotion of angiogenesis, and the development of resistance to cancer therapies^39,40^. Similarly, *MDK* and *IGF* signaling pathways have also been shown to play significant roles in the development and progression of numerous cancers^41–44^.

## Discussion

Vestibular schwannoma is a benign tumor with a low risk of metastasis or malignant transformation, but its growth can be unpredictable, potentially leading to severe complications, such as brainstem compression, and even death^45^. The underlying factors that drive the progression of vestibular schwannoma and lead to poor clinical outcomes remain poorly understood^46^. Our analysis of vestibular schwannoma samples uncovered distinct clusters and subpopulations of various cell types, along with their molecular signatures, which characterize their specific spatial locations within the tumor. During vestibular schwannoma progression, a distinct type of Schwann cells was identified, marked by overexpression of *VEGFA*, which showed enhanced abilities in proliferation, migration, and differentiation, contributing to significant functional and morphological recovery. These cells were found to be centrally located within the tumor. Furthermore, our spatially resolved analysis of vestibular schwannoma provided new insights into the interactions between Schwann cells and stromal cells.

Recent studies have characterized vestibular schwannoma using scRNA-seq. In line with recent studies, we observed the variability within the Schwann cells of these tumors and the association between myeloid cell infiltration and tumor progression^11^. Chu et al. conducted an analysis on a cohort of three patient tumors using their single-cell RNA sequencing data to identify potential drug therapy targets^12^. Furthermore, Barrett et al. determined that vestibular schwannoma-associated tumor Schwann cells (VS-SCs) closely resemble Schwann cells in the context of peripheral nerve injury and these injury-like VS-SCs might drive tumor growth by recruiting myeloid cells^13^. In contrast to these studies, we annotated them according to characteristic genes, classifying them into five types: *NOV*^hi^ SC, *JUNB*^+^ SC, *PMP2*^+^ SC, *VEGFA*^+^ SC and *PRX*^+^ SC. And while these studies have enhanced our understanding of current treatment approaches for vestibular schwannoma, they have not addressed the spatial distribution of different cell types within vestibular schwannoma. Thus, employing more precise methods to unravel the complex immune microenvironment and Schwann cells heterogeneity of vestibular schwannoma is essential for formulating evidence-based treatment strategies. Combined with our pre-study, we applied scRNA-seq and spatial transcriptomics to construct a preliminary cellular and spatial atlas of vestibular schwannoma.

*VEGFA*^*+*^ SCs showed enrichment in terms related to angiogenesis and various cellular processes. *VEGFA*, a member of the *PDGF*/*VEGF* growth factor lineage, encodes a heparin-bound protein essential for the proliferation and migration of vascular endothelial cells, playing a critical role in both normal and pathological angiogenesis^47,48^. Moreover, it has neurotrophic properties that play a critical role in Schwann cells proliferation and nerve regeneration after injury. Previous studies have indicated that Schwann cells that overexpress *VEGFA* show enhanced proliferation, migration, and differentiation, likely attributable to the activation of the *VEGFR2*/*ERK* signaling pathway^49^. In our study, we identified a distinct subgroup known as *VEGFA*^+^ SCs, which aligns with our previous researches and several recent single-cell RNA sequencing studies on vestibular schwannomas^14^. Notably, we discovered for the first time that *VEGFA*^*+*^ SCs were predominantly located in the core region of VS. Huo et al. demonstrated that aggressively growing vestibular schwannomas had a higher proportion of *VEGFA*^*+*^ SCs (*S100B+* cells) than those with stable growth using multiplex immunofluorescence staining^14^. These findings underscore the significant role of *VEGFA*^*+*^ SCs in the pathology of vestibular schwannoma. Overall, these findings highlight the functional specificity of different Schwann cell subgroups in areas such as cell differentiation, development, metabolism, and vascular formation.

This study revealed distinct spatial distribution patterns of cell types and gene expression in vestibular schwannoma through spatial transcriptomics analysis. *VEGFA*^+^ SCs were concentrated in the core region in VS_S1 sample, likely involved in angiogenesis, while *PRX*^+^ SCs were located near fibroblasts, potentially offering support or neuroprotection. Furthermore, different Schwann cell subtypes displayed relatively independent spatial arrangements; for example, *PMP2*^+^ and *JUNB*^+^ SCs are positioned close to *PRX*^+^ SCs, whereas *VEGFA*^+^ SCs were farther from *PRX*^+^ cells. This spatial organization suggested that various Schwann cell subtypes had distinct roles in tumor growth, immune interactions, and vascular development. These findings underscored the spatial heterogeneity of gene expression in vestibular schwannoma, providing new insights into its pathological mechanism.

Previous studies have shown that the spatial and temporal dynamics of tumor microenvironment are key factors leading to tumor heterogeneity, which is closely related to tumor progression^50,51^. Our cell communication analysis based on the curated receptor-ligand pair database showed that fibroblast in vestibular schwannoma microenvironment mainly regulated the biological behavior of tumor cells through *PSAP*/*GPR37L1*, *MDK*/(*ITGA6* + *ITGB1*), *IGF2*/(*ITGA6* + ) and *MDK*/*LRP1* signaling axes. In response to cellular stress, *PSAP* triggers endocytosis or activates pro-survival signaling pathways via binding to *GPR37* and *GPR371* receptors^52,53^. Campana et al.^54^ identified that treating Schwann cells in culture with prosaposin can promote their survival during cellular stress and increase the synthesis of sulfatide, a marker of myelin production. Mice deficient in prosaposin exhibit severe central and peripheral hypomyelination and a lack of mature oligodendrocytes^55^. These findings indicate that *PSAP* exerts a protective role in myelinating glial cells but does not promote their proliferation^56^. However, the specific role of *PSAP* secreted by fibroblasts remains to be explored in future studies. *MDK* (Midkine) is a secreted protein whose expression is significantly elevated in a variety of cancers^57–59^. It is associated with several key processes in tumor development and progression, including tumor cell proliferation, transformation, epithelial-mesenchymal transition, angiogenesis, and mitogenesis^42^. The communication between Schwann cells and fibroblast supports the idea that growth factors from fibroblasts act on Schwann cells by binding to different receptors.

Nevertheless, our study has its limitations. A key limitation of this study is lack of normal vestibular nerves from VS patients as controls and the small cohort size (3 tumors for scRNA-seq and 2 for spatial transcriptomics) may limit the generalizability of identified cell subtypes, particularly for rare populations. For spatial transcriptomics, we only collected samples from two patients, which could result in significant variability owing to the considerable cellular heterogeneity among individuals. And our scRNA-seq and spatial transcriptomics-seq were not implemented using the same samples. Last, we did not perform animal experiments or in vitro cellular studies to further validate our findings. In the future, it is essential to conduct an independent comparison with a larger sample size to confirm our results and reduce any potential bias related to individual differences. While spatial transcriptomics revealed a *VEGFA*-enriched Schwann cell subtype in our VS cohort, the functional and clinical implications of this population remain speculative. Future studies should integrate longitudinal imaging with multi-region tumor sampling to determine whether *VEGFA*^+^ SC abundance correlates with progression. Additionally, in vitro models could test whether *VEGFA* overexpression drives angiogenesis or tumor cell proliferation. Such mechanistic insights would strengthen the therapeutic rationale for targeting *VEGFA* signaling in VS.

## Methods

### Ethics

Samples from patients with pathologically confirmed vestibular schwannoma at the department of Otolaryngology, Shanghai Sixth People’s Hospital Affiliated to Shanghai Jiao Tong University School of Medicine were used in the study. The study was approved by the Ethical Committees of Shanghai Sixth people’s Hospital (approval no. 2021-242-1). Written informed consent was obtained from all participants prior to tissue collection. Sampling and handling of any patient material were performed in accordance with the ethical principles of the Declaration of Helsinki.

### Samples collection

ScRNA-seq was conducted on the 3 vestibular schwannoma samples previously analyzed in our prior study^11^. Furthermore, spatial transcriptomic profiling was performed on 2 additional vestibular schwannoma specimens acquired during the surgery.

### Clinical information

Clinical characteristics encompassing patient demographics (age, sex), audiometric profiles, tumor dimensions, and anatomical localization are detailed in Supplementary Fig. 1 and Supplementary Tables 1 and 2.

### Samples preparation

Upon obtaining fresh and intact clinical tissue samples, they were immediately preserved in dedicated single-cell preservation solution (Sinotech Genomics Co., Ltd, JZ-SC-18101, China), maintained on ice at 0-4°C, and subjected to subsequent single-cell suspension preparation within 2 hours.

### Single-cell suspension preparation

The procedure began with sterilizing small scissors and forceps using 70% ethanol and UV irradiation for 15–30 minutes to ensure aseptic conditions. The tissue samples were then placed in a dish containing ice-cold PBS and rinsed repeatedly to remove surface fascia and blood clots, with all steps performed on ice to maintain tissue viability. Then cleaned tissues were transferred to a 1.5 mL centrifuge tube with 400 μL of ice-cold PBS and minced into 2–4 mm³ fragments using sterilized scissors, keeping the tube on ice. After adding 800 μL of ice-cold PBS, the mixture was centrifuged at 300 ×g for 5 minutes at 4°C, followed by careful supernatant removal. For enzymatic digestion, 3–4 mL of pre-warmed (37°C) digestion buffer (Sinotech Genomics Co., Ltd, JZ-SC-58207, China) was added, and the tissue fragments were transferred to a 15 mL tube using a wide-bore pipette tip. The tube was sealed with Parafilm and incubated at 37°C on a horizontal shaker (90–105 rpm) for 30–120 minutes, with enzyme composition and digestion time optimized empirically for tissue-specific requirements. Digestion was halted by adding complete cell culture medium at a 1:5 ratio, followed by gentle mixing and sequential filtration through 100 μm, 70 μm, and 40 μm cell strainers. The filtrate was centrifuged at 500 ×g for 8 minutes at room temperature, and the supernatant was discarded. Cells were resuspended in 1 mL PBS, and suspension quality was assessed microscopically before quantifying cell count and viability using standard methods.

### Single cell transcriptome capture, library construction and sequencing

Cells were first stained with fluorescent dyes Calcein AM (live cells) and Draq7 (dead cells) to assess viability and quantify cell concentration using the BD Rhapsody™ Scanner (BD Biosciences). Following this, cells were loaded into a BD Rhapsody™ micro-well cartridge as described by Fan et al.^60^, with cell-capture beads added in excess to ensure near-complete bead occupancy in microwells; unbound beads were subsequently removed by washing. After cell lysis with lysis buffer, mRNA was captured on beads via oligo-dT primers, and the beads were retrieved, washed, and processed for reverse transcription to generate cDNA. Single-cell cDNA libraries containing cell barcodes and unique molecular identifiers (UMIs) were amplified from the beads using the BD Rhapsody™ cDNA Kit (Cat. No. 633773) and BD Rhapsody™ Targeted mRNA & AbSeq Amplification Kit (Cat. No. 633801), following the manufacturer’s protocols. Finally, libraries were sequenced on an Illumina NovaSeq platform in paired-end 150 bp (PE150) mode.

### 10x scRNA-seq data analysis and data processing

All analyses were performed in R (v4.3.2) using the Seurat package (v5.0) for single-cell and spatial transcriptomic data processing. The Seurat pipeline was employed for quality control, normalization, and downstream bioinformatics analyses. Low-quality cells were filtered out based on the following criteria: a proportion of mitochondrial genes count >15%. Following quality control, the data were normalized using the LogNormalize method. The top 2,000 highly variable genes were identified and retained for dimensionality reduction. Principal Component Analysis (PCA) was performed on scaled UMI counts using these highly variable genes. The first 25 principal components (PCs) were selected for downstream clustering. Nonlinear dimensionality reduction was subsequently performed using Uniform Manifold Approximation and Projection (UMAP) to visualize cell clusters in two dimensions.

Clusters were annotated by identifying transcriptional markers via the FindAllMarkers function with the Wilcoxon rank-sum test. Marker genes were defined by thresholds of |log₂ fold change | > 0.5 and minimum percentage of expressing cells (min.pct) > 25%. The top cluster-specific markers were then visualized in heatmaps to assess distinct transcriptional profiles.

### Sample preparation for 10x Genomics visium spatial transcriptomics

The tissues used in this study were acquired from patients following surgical resection. They were then fixed with 10% formalin PBS and embedded in paraffin. For spatial transcriptomics, a 10X FFPE gene expression slide (Part Number 1000185, from 10X Genomics) was utilized. The gene expression data from these spatial transcriptomics slides were captured using the 10x Genomics Visium Spatial Gene Expression platform, which leverages mRNA-binding oligonucleotides endowed with spatial barcodes as the standard method for capturing gene expression data.

Following data collection, a thorough quality check and mapping of the raw sequencing reads were conducted. Space Ranger software (version 1.3) was employed to align the cleaned, demultiplexed reads with the UCSC human GRCH38 reference genome. For downstream analysis, the R programming environment was used, specifically employing the Seurat workflow after procuring the single-cell gene expression count matrix. To ensure consistency in aligning spots across samples from different individuals, the approach outlined in the scRNA-seq data section was followed. This involved the use of the FindIntegrationAnchors and SCTransform functions for data integration and normalization.

### Cell type decomposition

To spatially resolve cell-type distributions across histological structures, we performed reference-guided deconvolution of the ST data using our integrated scRNA-seq dataset as a cellular atlas. Through application of RCTD^25^, we systematically mapped cell-type signatures from the scRNA-seq reference to spatially resolve transcriptomic profiles.

### Gene ontology (GO)

In this study, the clusterProfiler program within the R package was employed to calculate gene enrichment for each GO term. This was primarily achieved by matching the differential genes of each cell group with the terms in GO database. To identify the terms with the highest enrichment of differentially expressed genes, the number of differentially expressed genes in each term was determined. The enrichment intensity of these GO terms was determined based on the calculated p-value.

### Spatial map of cell dependencies in vestibular schwannoma

We used MISTy’s implementation in mistyR (v.1.9.1) to estimate the importance of the abundance of each major cell type in explaining the abundance of the other major cell types^61^. A multi-view model using three different spatial contexts: (1) an intrinsic view that measures the relationships between the deconvolution estimations within a spot, (2) a juxta view that sums the observed deconvolution estimations of immediate neighbours (radius, five spots), and (3) a para view that weights the deconvolution estimations of more distant neighbours of each cell type (radius, 15 spots).

In all models we included two classes of spatially contextualized predictive views: an intrinsic (intra) and a local neighbourhood view (para). For the para view, the effective radius was determined as the mean distance to the nearest neighbors plus the standard deviation. The weights for each point were calculated using a Gaussian family distribution.

### Identification of spatial ecotypes in ST data

To identify compositional cluster ecotypes, we applied the ISCHIA (Identifying Spatial Co-occurrence in Healthy and InflAmed tissues; v.1.0.0.0) algorithm implemented^26^. The optimal number of clusters (*k*) was determined to be five, using the elbow method on the mean cluster variance plot over a range of *k* from 2–30. PCA was used for dimensionality reduction before inputting visium data into ISCHIA. The Composition.cluster() function was then applied to the PCA dimensions of the gene expression spots. This function grouped the spots into ten distinct clusters, each representing a unique cellular composition. These clusters, or “spatial ecotypes”, were identified based on the principle that functionally related cell types tend to co-occur in specific spatial patterns within the tumor microenvironment. Differential gene expression analysis between ecotypes was conducted using the FindAllMarkers () function.

### Cell-cell communication analysis

To systematically analyze intercellular communication networks, we employed the CellChat (v1.1.3)^31^ and CellChat v2^62^, following a structured computational workflow. We selected homologous human genes for further analyses. First, CellChat objects were initialized using the createCellChat function, integrating preprocessed single-cell RNA-seq data and annotated cell clusters. Interaction probabilities between ligand-receptor pairs were then computed with computeCommunProb to mitigate outlier effects, followed by pathway-level communication inference via computeCommunProbPathway. To quantify signaling dynamics, network centrality metrics were derived using netAnalysis_computeCentrality, while netAnalysis_contribution identified dominant ligand-receptor pairs driving specific pathways. Visualization of communication networks was achieved using built-in functions (netVisual_aggregate and netVisual_individual).

### Immunofluorescence staining

Tissues were fixed in formalin, embedded in paraffin, and sectioned at 5 μm thickness. Following deparaffinization through sequential incubation in xylene and rehydration via a graded alcohol series, the slides were subjected to antigen retrieval by heating in EDTA Unmasking Solution (pH 8.0) (Simuwubio, Shanghai, China) within a microwave oven. The slides were then washed three times in phosphate-buffered saline (PBS) for 3 minutes each. To block non-specific binding, tissues were incubated with 3% Bovine Serum Albumin (BSA) in PBS for 30 minutes at room temperature. Subsequently, the slides were incubated overnight at 4°C with primary antibodies directed against SPP1 (Affinity Biosciences Cat# AF0227, RRID:AB_2833402) and CD44 (Proteintech, Cat No. 60224-1-Ig). Following primary antibody incubation, the slides were incubated with appropriate secondary antibodies for 1 hour at room temperature in the dark. Finally, nuclei were counterstained using DAPI. The resulting images were processed and analyzed using ZEN 2011 software (Carl Zeiss, Jena, Germany).

## Supplementary information

Supplementary materials.

## Data Availability

The scRNA-seq data was downloaded from the National Omics Data Encyclopedia (https://www.biosino.org/node; accession code OEP001871). Further information is available from the corresponding author, Dongzhen Yu (email: 7250012023@shsmu.edu.cn).

## Code availability

The codes during the current study are available from the corresponding author.

## Abbreviations

DEGs: differentially expressed genes
H&E: Hematoxylin and eosin
SC: Schwann cell
ST: Spatial transcriptomes
UMAP: Uniform Manifold Approximation and Projection
VS: vestibular schwannoma
