# Supplementary material for: Integrating single-cell and spatial transcriptomics reveals the cellular heterogeneity of vestibular schwannoma
Source: NPJ Precis Oncol. 2025 Jul 8;9:228. doi: 10.1038/s41698-025-01028-y (PMC12238636; doi:10.1038/s41698-025-01028-y)
Supplement: Supplementary file 1 — Supplementary materials. [file 41698_2025_1028_MOESM1_ESM.pdf]

Supplemental Material
1. Supplementary Figures
2. Supplementary Tables

1. Supplementary Figures

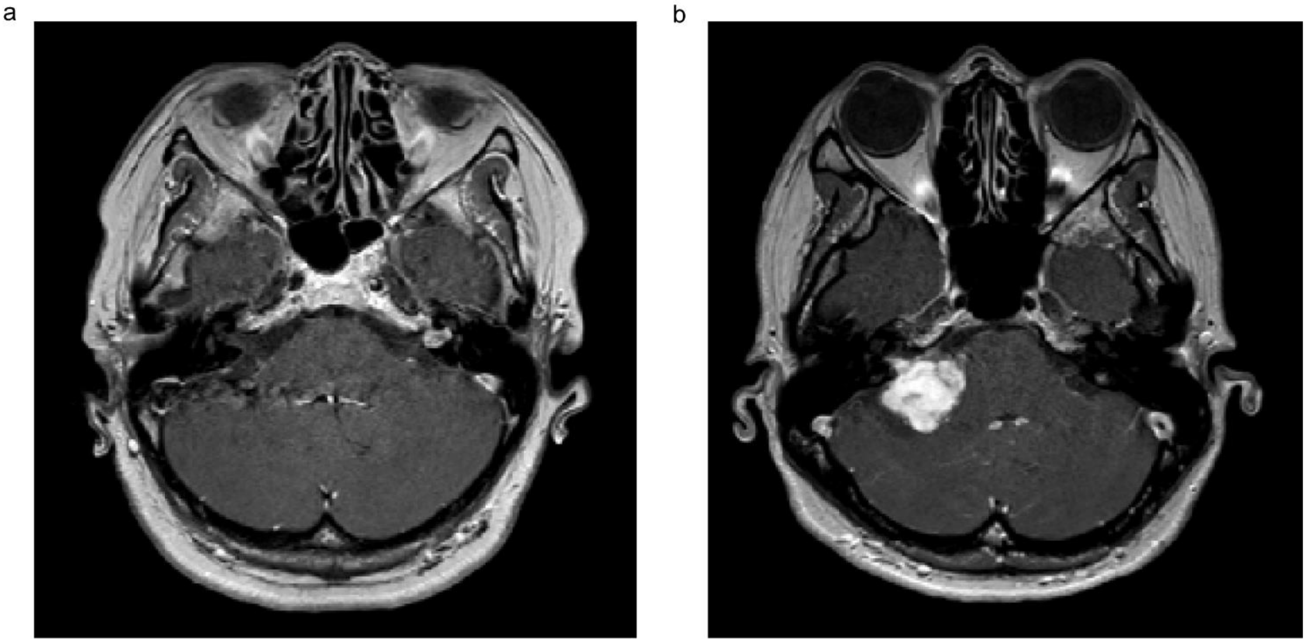

Supplementary Fig. 1 Magnetic Resonance Imaging (MRI) characteristics of study participants with vestibular schwannoma. a-b MRI images of patients (VS_S1-2) with vestibular schwannoma obtained from the spatial transcriptome study.

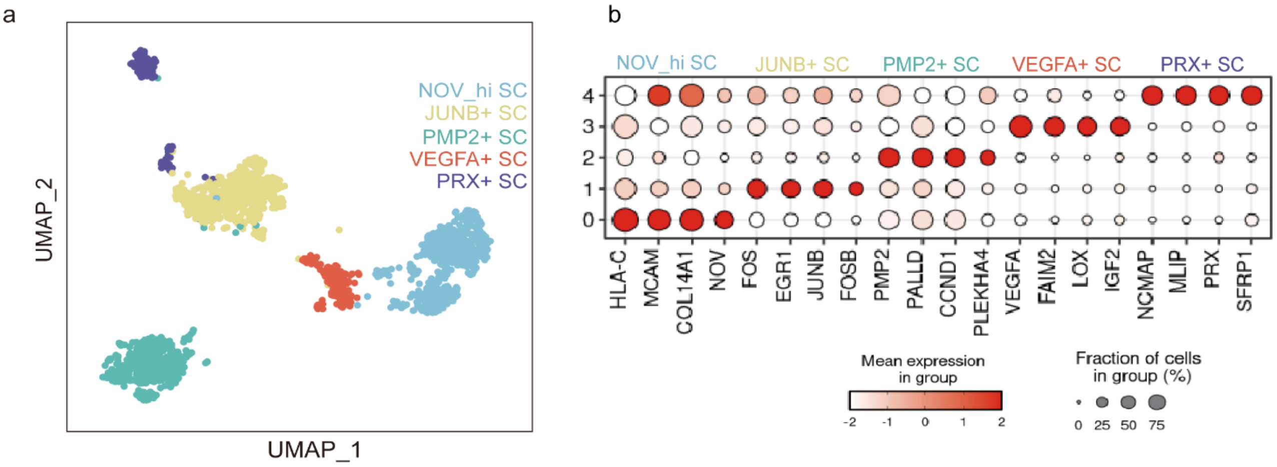

Supplementary Fig. 2 Schwann cells sub-clustering: *NOV*^{hi} SC, *JUNB*$^{+}$ SC, *PMP2*$^{+}$ SC, *VEGFA*$^{+}$ SC, and *PRX*$^{+}$ SC. a UMAP visualization of 5 subclusters of Schwann cells from integrated vestibular schwannoma data. **b** Dot plot of top four markers in five clusters.

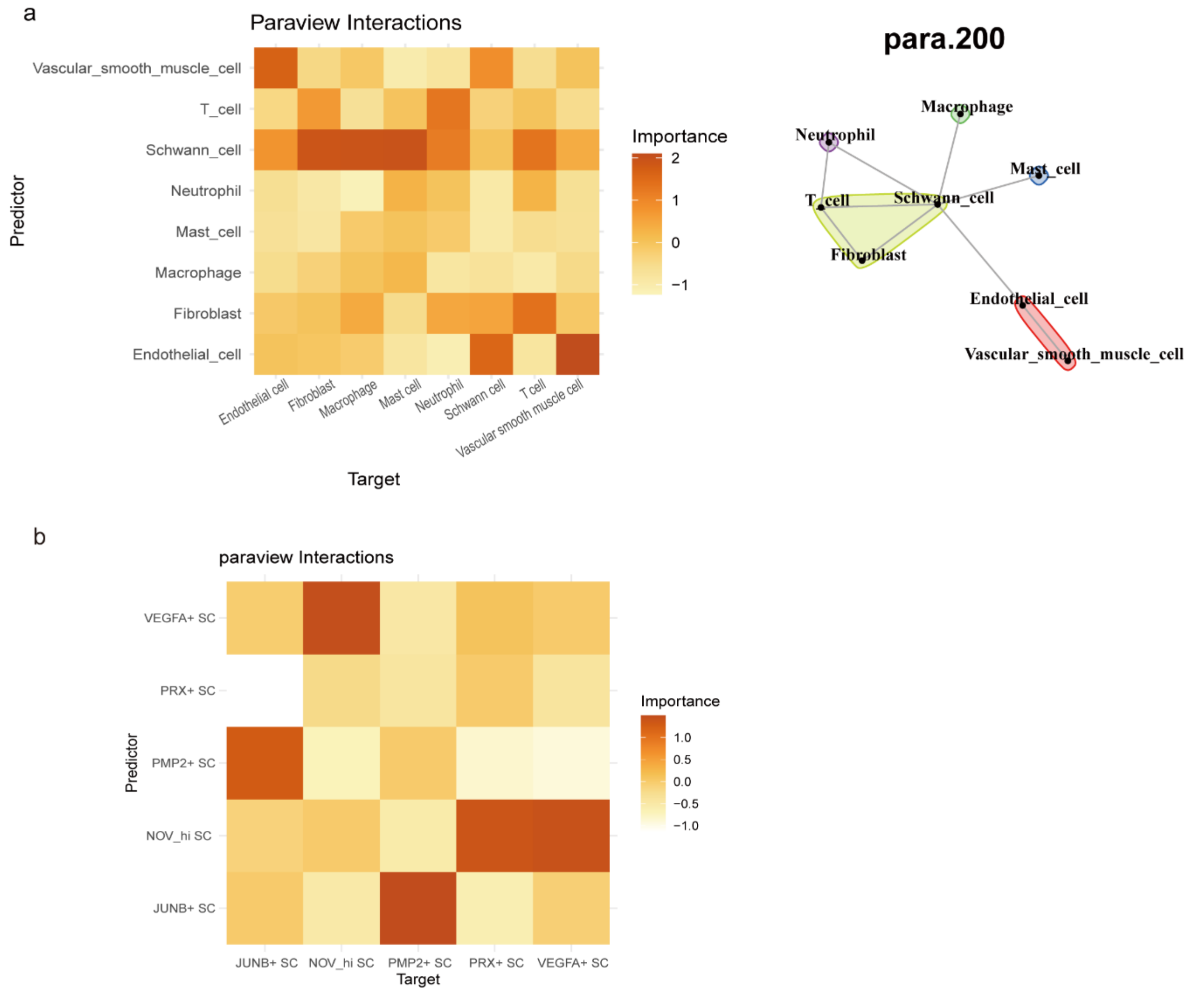

Supplementary Fig. 3 Characterization of cell types abundance. a Median importance of the abundance of major cell types within the local neighbourhood (effective radius of 5 spots) in spatial transcriptomics (left). Schematic representation of paraview cell–cell interaction networks using MISTy within the local neighbourhood (right). **b** Median importance of the abundance of major cell types within the local neighbourhood in SC subtypes.

2. Supplementary Tables

Supplementary Table 1. Clinical information of the patients with VS in our previous datasets

	Sample ID	Sex	Age	Side	Size (cm)	Cystic degeneration	Mean audiometric threshold (dB HL)	Koos classification
Xu M et al.	VS1	F	66	R	1.6×1.3	SVS	Left:15 Right:70	Grade II
	VS2	M	52	R	16×1.2	SVS	Left:10 Right:57	Grade II
	VS3	F	31	L	15×1.5	SVS	Left:63 Right:30	Grade II

M, male; F, female; L, left; R, right; SVS, solid vestibular schwannoma; CVS, cystic vestibular schwannoma.

Supplementary Table 2. Clinical information of the patients with VS obtained from ST

Sample ID	Sex	Age	Side	Size (cm)	Cystic degeneration	Mean audiometric threshold (dB HL)	Koos classification
VS_S1	F	59	L	0.9× 0.5	CVS	Left:58 Right:33	Grade I
VS_S2	F	47	R	2.9× 2.8	SVS	Left:7 Right:>90	Grade III

M, male; F, female; L, left; R, right; SVS, solid vestibular schwannoma; CVS, cystic vestibular schwannoma.
